# Highly Stretchable Fully Biomass Autonomic Self-Healing Polyamide Elastomers and Their Foam for Selective Oil Absorption

**DOI:** 10.3390/polym13183089

**Published:** 2021-09-13

**Authors:** Palraj Ranganathan, Chin-Wen Chen, Syang-Peng Rwei

**Affiliations:** Research and Development Center of Smart Textile Technology, Institute of Organic and Polymeric Materials, National Taipei University of Technology, No. 1, Sec. 3, Chung-Hsiao East Road, Taipei 10608, Taiwan; rangapalraj@gmail.com

**Keywords:** polyamide elastomers, highly stretchable, self-healing, hydrophobic foam, oil absorption

## Abstract

Renewable polymers with self-healing ability, excellent elongation, hydrophobicity, and selective oil absorption attributes are of interest for an extensive range of applications, such as e-skin, soft robots, wearable devices, and cleaning up oil spills. Herein, two fully renewable eco-friendly polyamide (PA)-based self-healing elastomers (namely, PA36,IA, and PA36,36) were prepared by a facile and green one-pot melt polycondensation of itaconic acid (IA), Pripol^TM^ 1009, and Priamine^TM^ 1075 monomers. The molecular structures of these PAs were analyzed by FITR, ^1^H NMR, and ^13^C NMR. The distinct structure of these PAs shows superior strain values (above 2300%) and high ambient temperature autonomous self-healing ability. Interestingly, the synthesized renewable PA36,36 showed zero water absorption values and hydrophobic properties with a contact angle of θ = 91° compared to the synthesized PA36,IA and other previously reported PAs. These excellent attributes are due to the low concentration of amide groups, the highly entangled main chains, the intermolecular diffusion, the manifold dangling chains, and the numerous reversible physical bonds within the renewable PAs. Furthermore, the hydrophobic properties may aid in the selective oil absorption of the PA36,36-based foam, for which PA36,36 foam is produced by the green supercritical carbon dioxide (scCO_2_) batch foaming process. The PA36,36 foam with a microporous cellular structure showed better absorption capacity and high stability in repeated use. Due to these advantages, these bio-based PAs have potential for the production of eco-friendly self-healing materials, superabsorbent foams, and other polymeric materials.

## 1. Introduction

Recently, active interest in bio-based polymeric materials has increased, making the innovation of bio-based plastics one of the most fascinating fields in materials science [1]. Bio-based polymers are prepared from monomers derived from biomass feedstock [2,3]. Compared to fossil fuel-based commodity polymers, the use of biomass polymers results in the reduction in carbon dioxide (CO_2_) emissions into the atmosphere and oil consumption [4]. Therefore, the growth in these kinds of sustainable polymers not only alleviates the over-reliance on petroleum feedstock, but also offers new products with a notably improved ecological footprint and performance compared to their fossil-based counterparts [5].

Service life and implementation are two vital factors affecting the practical applications of bio-based polymers. Currently, the self-healing effect is considered one of the most effective methods to extend the life of bio-based polymers. In recent decades, remarkable advances have been made in the development of self-healing bio-based polymer elastomers [6,7]. Elastomers are one of the most studied self-healing polymer materials due to their versatile applications, such as electronic devices, coatings, sealings, clothing, medical devices, tires, and daily commodities [8,9,10]. The name “elastomer” is derived from rubber, which is typically defined as a highly stretchable material that can retract forcibly and quickly to maintain its original dimensions while releasing considerable force. Elastomers are viscoelastic polymers (i.e., characterized by both elasticity and viscosity), having weak intermolecular forces, commonly high failure strain, and low Young’s modulus compared to other materials. Rubber is an elastic material derived from natural and petroleum gas (synthetic) or acquired from the exudations of some tropical plants (natural rubbers). Elastomers and rubbers are commonly used terms to refer to any materials with rubber-like attributes [11].

A milestone study in self-healable elastomer systems was documented in 2008 [12]. Subsequently, healable elastomer materials have attracted considerable attention because they can fulfil the needs associated with sustainable growth [13]. The excellent characteristics of self-healing elastomer materials create prospects for their use in high-end applications, such as wearable devices, electronic skin/smart flexible electronics, and soft robots [14,15,16,17,18]. Specifically, the properties of autonomous self-healing elastomers are preferable because they may self-heal without any exterior intervention after experiencing damage. Self-healing capability elastomers are activated by physical bonds such as van der Waals forces [19], ionic interaction [20], hydrogen bonds [21], host–guest interaction [22], metal–ligand interaction [23], the combination of various bonds [24], covalent bonds such as the disulfide bond [25], the Diels Alder reaction [26], and boronic oxide/ester [27].

However, the integration of most autonomous self-healing elastomeric materials includes multiple steps using noxious compounds, which are undesirable for green polymer production, create challenges for scaling up production, and are not cost effective [28]. Furthermore, few fully bio-based autonomous self-healing elastomeric materials have been reported to date; published studies involve bio-based instances that are not 100% bio-based or are partially bio-based [29,30,31]. The most general method employed in the polymer processing industry is the melting process, which is eco-friendly. Solution polymerization based on acid chlorides/dimethyl esters results in greater toxic condensates and the raw materials utilized in this kind of process require additional synthetic steps and work-up. Additionally, toxic solvents are required to remove the homogeneous catalyst after manufacturing [32]. Therefore, solvent-free melting polycondensation is one of the most extensively utilized synthetic methods for the production of commercial engineering plastics such as polyamides (PAs), polycarbonates (PCs), and polyesters (PEs) [33,34]. In this melting polymerization, monomers are melted and subjected to a polycondensation reaction to yield a highly viscous resin, which can be directly processed into the desired materials. In 1946, Whinfield first developed a melting polymerization path for polyester production by the transesterification polycondensation of diols with diester monomers [35]. This method is still used for the production of millions of tons of polyester each year. Moreover, the preparation of healable elastomers via solvent-free melting polymerization remains a vitally important process. Therefore, interest in self-healing elastomer research aimed at green, eco-friendly, and energy-efficient manufacturing has increased due to the scarcity of petroleum-based resources and significant concerns related to the effect of greenhouse emissions [33].

In addition, significant attention has also focused on using hydrophobic self-healable polymer foams to produce oil absorbents due to their facile, rapid, and effectual absorption and oil/water separation capability [35,36]. This is motivated by the large-scale oil spills that have occurred in line with the development of modern industry, which cause acute harm to local ecosystems. For instance, more than 600 crude oil barrels spilled into a river in Santander, Colombia, in March 2018, leading to a catastrophe for the local ecological system [37]. To assist with this issue, many innovative polymeric porous materials with superhydrophobic properties have been developed, including microspheres [38], aerogels [39], membranes [40], and monoliths [41], which can separate oil from water. Nonetheless, most research studies have the following issues. First, the manufacturing process of these products is highly complex and costly, which prevents their large-scale use. Second, most of the previously mentioned products are composites, thus creating issues in the recycling of the products after their use. Ultimately, modern industrial manufacture faces the issue of scarcity of fossil-based non-renewable resources [37]. Hence, it is considered to be highly attractive to develop a pristine fully bio-based polymeric elastomer with multifunctional characteristics, such as autonomous self-healing abilities and oil/water separation properties, which can be easily manufactured and are derived from green resources.

In this study, we report on the development of two classes of fully biomass-derived polyamide (PA)-based self-healing thermoplastic elastomers via a green melt-polycondensation approach without the use of solvents and additives, using bio-based monomers (itaconic acid (IA) derived from the fermentation of glucose, and Pripol^TM^ 1009 and Priamine^TM^ 1075 derived from natural fatty acids). Both Pripol^TM^ 1009 and Priamine^TM^ 1075 contain four methylene chains with a total of 36 carbon atoms. Due to the numerous methylene dangling chains and long methylene main chains present in Pripol^TM^ 1009 and Priamine^TM^ 1075 monomers, it is thought that robust physical and reversible cross-links can be formed between the methylene chains via van der Waals forces. This produces highly stretchable and autonomous self-healing polyamide elastomers. Interestingly, PA36,36 exhibits zero water uptake properties due to the significant content of non-polar parts. Therefore, the foams were prepared via a green supercritical carbon dioxide (scCO_2_) process using this hydrophobic PA36,36 elastomer. As a proof of concept, the use of hydrophobic PA36,36 foams to effectively eliminate oil from water was proven.

## 2. Experimental Section

### 2.1. Materials

The fatty dimer diamine under the tradename Priamine^TM^ 1075 (molecular weight ~570; ~99% dimer diamine content; low viscosity liquid, amine value 205 mgKOH/g, renewable carbon content 100%) and fatty dimer diacid Pripol^TM^ 1009 (molecular weight ~570; ~98.7% dicarboxylic acid and ~1.0% tricarboxylic acid; acid value 196 mg·g^−1^, and iodine value 4.5 g/100 g; renewable carbon content 100%) were purchased from Croda (Netherland) International Plc. Itaconic acid (IA) was procured from Sigma Aldrich (St. Louis, MO, USA). Chloroform-d (CDCl_3_, ≥99.8%), deuterium oxide (D_2_O), and tetrahydrofuran (THF, 99.9%, HPLC grade) were purchased from Sigma Aldrich and used as received. The motor oil and n-hexane oil employed for the absorption experiment was procured from Sigma Aldrich.

### 2.2. Synthesis of PA36,IA Thermoplastic Elastomer

**Scheme 1:**
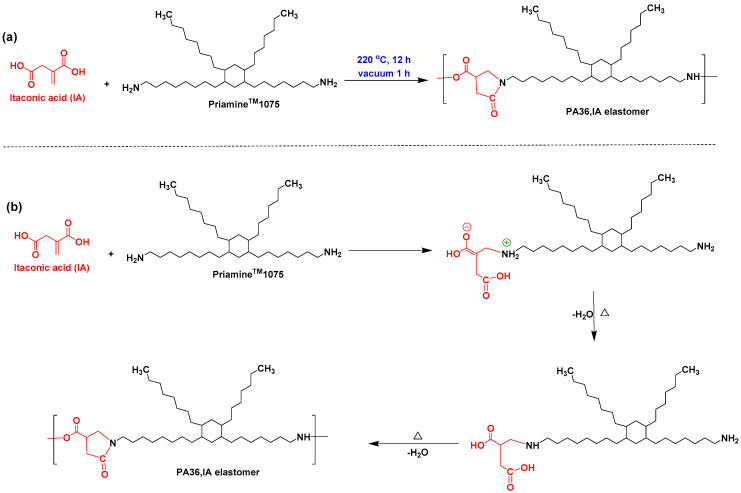
(**a**) The synthesis procedure of PA36,IA elastomer and (**b**) the formation mechanism of PA36,IA derived from IA and Priamine^TM^ 1075. Note: Priamine^TM^ 1075 is a derivative of natural fatty acids, mostly composed of alicyclic diamines; at least 98.7% of the Priamine^TM^ 1075 is diamine-functional.

The fully bio-based PA36,IA elastomer was synthesized via green one-step melt-polymerization under a nitrogen gas environment without the use of solvents or additives. The polymerization reactor used in this work is shown in Figure S1 (Supplementary Material). A typical synthetic procedure is described as follows (Scheme 1a): IA (1000 mmol, 130 g) and Priamine^TM^ 1075 (1000 mmol, 570 g) were weighed and introduced into a 2 L polymer reactor equipped with a central mechanical stirrer, a nitrogen gas inlet and outlet, a distillation setup thermometer, and a vacuum pump connection. To prevent excess foaming or bubbles, the reaction temperature was gradually increased by 20 °C each 25 min until attaining 220 °C. Subsequently, the polymerization was performed for 12 h at 220 °C. After 12 h, the polymerization reaction was continued for 1 h under reduced pressure (P = 0.7 kPa). The final product was transferred to a storage container from the reactor. The yield of the final product reached ~97%. The synthesis of PA36,IA was performed by polycondensation following an aza-Michael addition reaction between the IA and the Priamine^TM^ 1075.

**Scheme 2:**
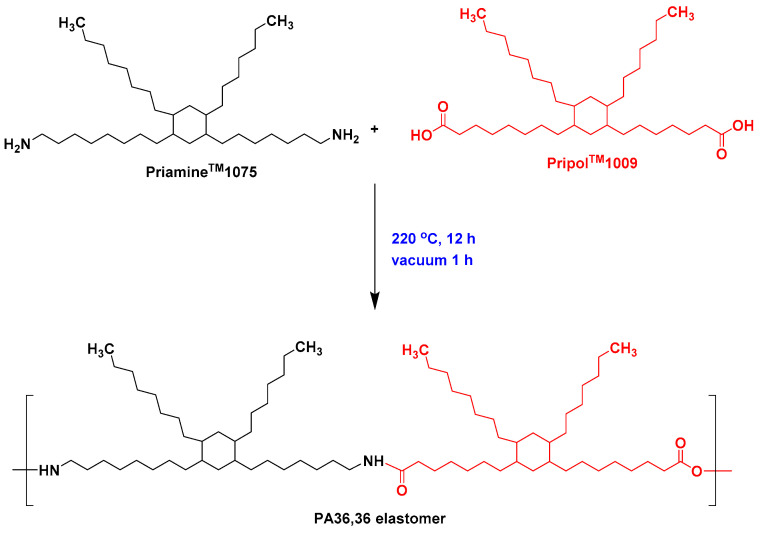
The synthesis procedure of PA36,36 elastomer. Note: Pripol^TM^ 1009 is derived from natural fatty acids, mostly composed of an alicyclic dicarboxylic acid; at least 98.8% of the Pripol^TM^ 1009 is dicarboxylic acid-functional.

A schematic of the polycondensation reaction between Priamine^TM^ 1075 and IA is shown in Scheme 1b. During the polymerization (at 220 °C), solitary electrons in the primary amine group of Priamine^TM^ 1075 nucleophilically attacked the double bond of IA in the β state to create an enolate intermediate. The protonation of enolate intermediate produced the new unsaturated carbonyl compound. Then, the amide polycondensation reaction continuously increased the polymer chain and formed an IA chain from the extended PA36,IA elastomer with a pyrrolidone ring structure [42]. The same procedure and reaction conditions were followed for the synthesis of PA36,36 (Scheme 2), and the detailed procedure is provided in the Supplementary Material.

### 2.3. Supercritical Carbon Dioxide (scCO_2_) Batch Foaming Process

The PA36,36 foam was prepared by a batch foaming process employing scCO_2_ as a green blowing agent. Firstly, the PA36,36 sample (5 cm × 1 cm × 5 cm) was placed into a high-pressure chamber at a constant temperature of 50 °C and saturation pressure of 150 bar. The foaming process was maintained for 1 h. Thereafter, the pressure was abruptly reduced by releasing scCO_2_ at 150 bar to 0.1 bar within 3 s to provide the driving force for cell growth and nucleation. Finally, PA36,36 foam was fabricated.

### 2.4. Characterization

Fourier transform infrared (FTIR) spectra of PAs were recorded with a Nicolet 5700 (Waltham, MA/Madison, WI, USA) FT-IR spectrometer using a KBr pellet technique with a resolution of 1 cm^−1^ over a scan range of 4000–500 cm^−1^. The number of co-added scans was 62. The transparency of the PAs was investigated by UV-Visible spectra in the film state (thickness ~40 µm). The optical transmittance UV-Visible spectra were recorded on a Shimadzu UV 3101-PC spectrophotometer at ambient temperature.

The proton and carbon environment of PAs was determined by a ^1^H NMR and ^13^C NMR Bruker AVANCE-III 400 MHz spectrometer (Billerica, MA, USA) using CDCl_3_-d and D_2_O-d as the solvents. Data was obtained using MestReNova software and the chemical shifts are stated in ppm relative to the tetramethylsilane (TMS) standard.

The number-average molecular weight (M_n_), polydispersity index (Ð), and weight-average molecular weight (M_w_) of all of the PA elastomer materials were evaluated by gel permeation chromatography (GPC, JASCO RI-2031). THF was utilized as an eluent with a 0.75 mL/min flow rate. Polymer samples were prepared at a concentration of 2 mg mL^−1^ in THF eluent. Linear polystyrene was employed as a standard for calibration. A refractive detector index was operated at 25 °C.

Thermal gravimetric analysis (TGA) and derivative thermogravimetry (DTG) analysis were carried out with a Q50 TGA (TA Instrument, Newcastle, DE, USA) in the presence of a nitrogen gas flow at a flow rate of 50 mL min^−1^. The weight loss or thermal behavior of each PA elastomer (8–10 mg) were collected by heating the PA samples at a constant rate of 10 °C min^−1^ over the temperature range of 30–800 °C.

Differential scanning calorimetry (DSC) measurements were performed with a SHIMADZU AGS-X 500 N DSC analyzer (Waltham, MA, USA) under a nitrogen gas atmosphere. Approximately 5–8 mg of dried sample (dried at 50 °C overnight under vacuum) was weighed and sealed into an aluminum pan. All of the samples were heated from 0 to 200 °C and then cooled from 200 to 0 °C, at a heating/cooling rate of 5 °C min^−1^.

Dynamic mechanical analysis (DMA) of the PAs was performed on a DMA Q800 (TA Instrument, Newcastle, DE, USA) model equipped with a cooling liquid-nitrogen apparatus in compression mode. Each PA sample with a thickness of 5 mm and a diameter of 7 mm was tested from −70 to 80 °C at a 1 Hz frequency with a deformation amplitude of 3% and a heating rate of 3 °C min^−1^. The T_g_ of the PAs were created from the tan δ peak curves, which are the ratio of storage (G’) and loss (G”) moduli.

Tensile attributes were measured using an INSTRON 3800R (Norwood, MA, USA) universal testing machine with the ASTM D638 standard. Dumbbell-shaped samples were made by injection molding. The molding process was conducted with the following conditions: molding temperature, 200 °C; injection flow, 90 cm^3^/s; injection pressure, 500 bar; and holding time 30 s. The tensile analysis was conducted at room temperature with a crosshead speed of 50 mm min^−1^. Each test was repeated six times and the average standard deviations were calculated. A self-healing test was performed by cutting the original polymers into two parts with a razor blade. The separated polymers were then immediately reconnected by hand for 1 min at ambient temperature and left for various periods. Healing efficiency was calculated by the ratio of the strain between the original polymer and the healed polymer sample. Five samples were tested for each polymer.

Surface analysis of the self-healed PAs (cracks before and after self-healing) was examined using a Veeco NT9100 polarizing optical microscope (POM, BX-51, Shanghai Wumo Optical) equipped with a CCD camera at 25 °C. A Hitachi Tabletop Microscope TM 4000 Plus scanning electron microscope (SEM) was used at 12.8 mm × 500 magnification and 15 kV accelerating voltage to inspect the surface of self-healed elastomers (cracks before and after self-healing).

The rheological attributes of the PAs were scrutinized using an Anton Paar Physica MCR-301 rheometer equipped with 25 mm cone-plate geometries. The outcomes of loss modulus, storage modulus, loss factor, and complex viscosity at an angular frequency from 0.01 to 628 rad/s were obtained at a strain amplitude of 0.5%. Measurements were carried out at 150 °C.

Water absorption of PA samples (dimensions of 3 cm × 2 cm × 3 cm) was attained by assessing their weights before and after soaking in water at 25 °C for 24 h. Following determination of water absorption, PA elastomers were moderately dried with a tissue to eliminate water from the PA materials’ surface. Five samples were tested for each PA.

(1) $$\mathrm{Water}\;\mathrm{absorption}\;\left(\%\right)=\frac{W_{\mathrm{wet}}-W_{\mathrm{dry}}}{W_{\mathrm{dry}}}\;\times \;100$$

Contact angle analysis was performed with sessile drop using a Biolin Surface Tensiometer. A square film of PA material was positioned on the glass slide top. Then, a drop of 10 µL of water was positioned on top of the elastomer film surface with a 50 µL syringe. Six different tests were performed on diverse spots of elastomer specimens. The contact angle was attained from the mean of the right and left angles of the water droplets.

The cellular morphology of PA36,36 foam was examined on a Hitachi Tabletop Microscope TM 4000 Plus scanning electron microscope (SEM) at a magnification of 12.8 mm × 500 and 15 kV accelerating voltage.

The oil absorption capacity of the PA36,36 foam was tested by dipping foam in oils (n-hexane or motor oil) for 10 min and weighing. The absorption capacity of the PA36,36 foam was calculated as follows:

(2) $$Q=\frac{m_{t}}{m_{0}}-1$$

where *m_t_* and *m*_0_ are the weight of the oil-absorbed sample and the initial sample, respectively.

The reusability of PA36,36 foam was measured by the absorption-desorption method. First, PA36,36 foams were saturated with oils (hexane or motor oils), and then the oils were eliminated by centrifugation for 20 min. The absorption-desorption test was repeated 5 times in the same foam sample to examine the alteration in the capacity of absorption in multiple cycles.

## 3. Results and Discussion

### 3.1. Design, Synthesis, and Structure Characterization of the Bio-Based Thermoplastic PA Elastomers

**Figure 1:**
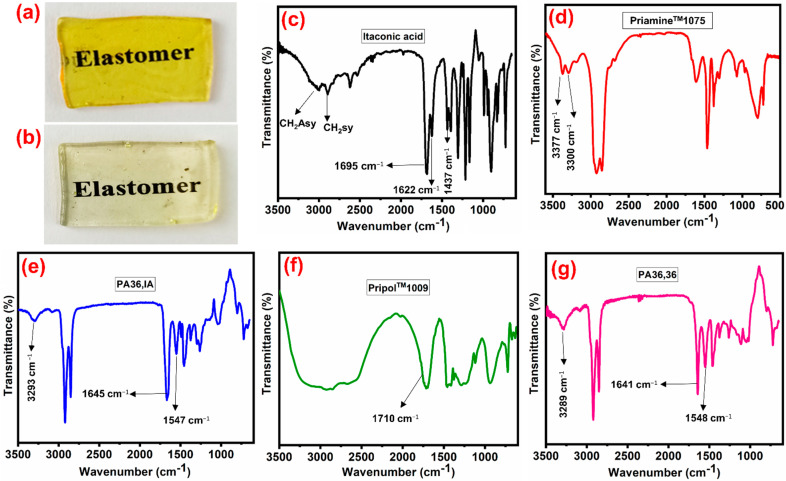
Photographic images of transparent PAs: (**a**) PA36,IA and (**b**) PA36,36; FTIR spectra of (**c**) IA, (**d**) Priamine^TM^ 1075, (**e**) PA36,IA, (**f**) Pripol^TM^ 1009, and (**g**) PA36,36.

The prime aim of the present work was to synthesize and characterize two types of fully bio-based PA elastomer derived from renewable monomers, and to apply them to self-healing and oil absorption applications. The bio-based PAs, namely PA36,IA and PA36,36, were synthesized by the polycondensation of Priamine^TM^ 1075 with IA or Pripol^TM^ 1009 at the temperature of 220 °C (Scheme 1 and Scheme 2), respectively. Melt-polycondensation was used to diminish the ecological effect of the synthesis of the preferred PAs. The bio-based PAs showed a slightly yellow transparent appearance (Figure 1a,b) and high elongation. The transformation of monomers into bio-based PAs was confirmed by FTIR, ^1^H NMR, and ^13^C NMR spectroscopy.

Figure 1c–e shows the FTIR spectra of bio-based PA36,IA, and its monomers. The IR band in the regions of 1437, 1622, and 1695 cm^−1^ are associated with the =CH_2_ scissoring bending vibration, and C=O and C=C stretching vibrations, respectively, and they are allocated to the chemical structure of the IA monomer (Figure 1c). In the FTIR spectrum of Priamine^TM^ 1075 (Figure 1d), the bands at 3377 and 3300 cm^−1^ related to the stretching vibration of the primary amino group. The band in the region at 3300 cm^−1^ is related to the secondary amino group of Priamine^TM^ 1075 [43]. However, in the FTIR spectrum of PA36,IA (Figure 1e), primary and secondary amino group peaks (3377 and 3300 cm^−1^) were not observed and a new broad single absorption peak appeared in the region at 3293 cm^−1^. This peak is assigned to the amide group (-NH-) in PA36,IA, which indicates that a successful polycondensation reaction took place between IA and Priamine^TM^1075. The single absorption band at 3293 cm^−1^ also represents the strongest H-bond between the PA amide groups. The apparent difference between the bands of the carbonyl groups in IA (Figure 1c) and PA36,IA (Figure 1e) can be witnessed in the FTIR spectra. The characteristic carbonyl band of IA at 1695 cm^−1^ has almost completely disappeared, and the carbonyl bands of amide-I (1645 cm^−1^) and amide-II (1547 cm^−1^) were raised in the PA36,IA FTIR spectrum (Figure 1e), respectively [42]. These outcomes indicate the conversion of the monomers into bio-based PA36,IA.

For PA36,36 (Figure 1g), the existence of the amide bond was detected in several wavenumber regions: at 3289 cm^−1^ (N–H stretching in amide-I); 1641 cm^−1^ (C=O stretching in amide-II); 1548 cm^−1^ (in-plane N–H bending coupled with C–O, and C–N stretching in amide-III); and 1261 cm^−1^ (N–H bending in amide-IV), indicated the reaction between Priamine^TM^ 1075 and Pripol^TM^ 1009 monomers. The disappearance of the band at 1710 cm^−1^ (the C=O stretching of carboxylic acid) (Figure 1f) confirmed that the Pripol^TM^ 1009 had been completely amidized [44]. The transparency of the PAs was supported by the outcomes from the UV-Vis spectrophotometer (Figure S2, Supplementary Material), which evidence the high optical transparency.

**Figure 2:**
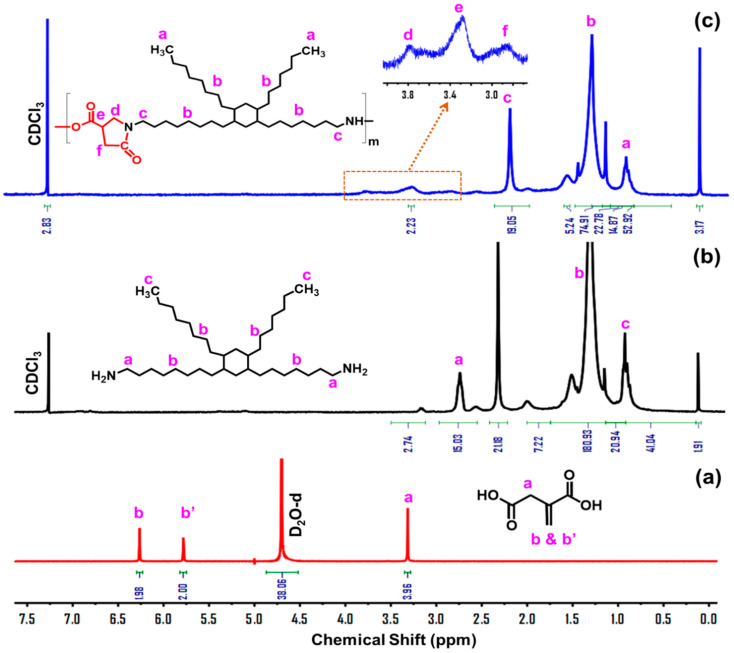
^1^H NMR spectra of (**a**) IA, (**b**) Priamine^TM^ 1075, and (**c**) PA36,IA.

The ^1^H NMR spectra of IA, Priamine^TM^ 1075, and PA36,IA are presented in Figure 2. The chemical shifts at 3.32, 5.77, and 6.26 ppm are allocated to the hydrogen atoms of the IA (Figure 2a). The chemical shift of proton in the CH_2_ adjacent to NH_2_ was witnessed at 2.67 ppm for the Priamine^TM^ 1075. The peaks in the region at 1.00–1.52 ppm are assigned to the –CH_2_ protons in the middle of the Priamine^TM^ 1075 chains. The peak at 0.89 ppm is related to the –CH_3_ protons at the end of the Priamine^TM^ 1075 dangling chains (Figure 2b). In the ^1^H NMR spectra of PA36,IA (Figure 2c), the chemical shift at 0.89 ppm, and at 1.00–1.52 ppm, are attributed to the aliphatic –CH_2_ protons of the PA36,IA segments, whereas the chemical shifts at 2.21 ppm represent the –CH_2_ proton adjacent to the pyrrolidone ring. The chemical shifts in the region at 2.91, 3.29, and 3.77 ppm refer to the protons of the pyrrolidone ring, i.e., the creation of pyrrolidone ring [42]. These results further support the successful synthesis of the PA36,IA elastomer.

**Figure 3:**
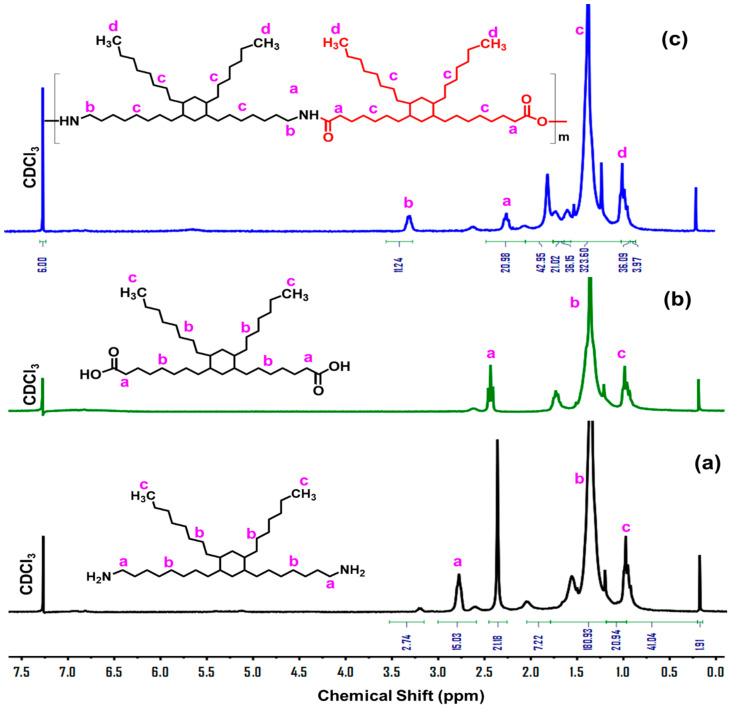
^1^H NMR spectra of (**a**) Priamine^TM^ 1075, (**b**) Pripol^TM^ 1009, and (**c**) PA36,36.

In the ^1^H NMR spectrum of Priamine^TM^ 1075 and Pripol^TM^ 1009 (Figure 3a,b), the chemical shift of Pripol^TM^ 1009 is similar to that of Priamine^TM^ 1075, with the exception that the chemical shift of proton in the –CH_2_ adjacent to –NH_2_ was witnessed at 2.67 ppm for Priamine^TM^1075, and the proton of the CH_2_ adjacent to –COOH was witnessed at 2.36 ppm for Pripol^TM^1009. For PA36,36 (Figure 3c), the –CH_2_ protons adjacent to the –CO and –NH, which signify the amide linkage, were detected at 2.17 and 3.26 ppm, respectively [45]. The chemical structures of PA36,IA, and PA36,36 were further supported by the results of ^13^C NMR (Figure S3, Supplementary Material), which show the chemical shift for the PAs, providing evidence of the –CO groups and the alkyl dangling chains.

**Figure 4:**
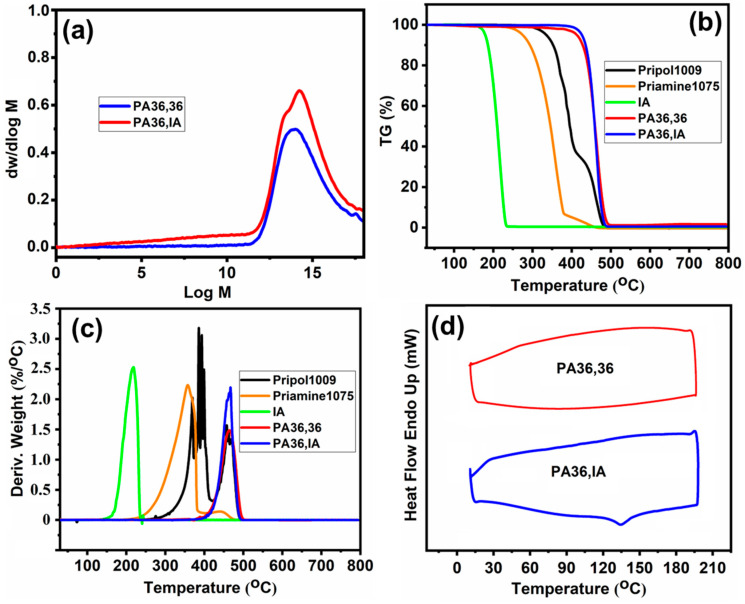
(**a**) GPC, (**b**) TGA, (**c**) DTG, and (**d**) DSC traces for the synthesized PAs.

The resulting PAs were then analyzed by GPC (Figure 4a) and the relevant information is presented in Table 1. The outcomes display that the synthesis led to PA36,IA with M_n_, M_w_, and Ð values of 19,262 g mol^−1^, 30,889 g mol^−1^, and 1.60, respectively. For PA36,36, the M_n_, M_w_, and Ð values were 21,045 g mol^−1^, 32,938 g mol^−1^, and 1.57, respectively. The greater reactivity of Pripol^TM^ 1009 may be a factor in the M_w_ gain.

### 3.2. Thermal Properties

TGA analysis was performed to study the thermal decomposition behavior of bio-based PA elastomers. Figure 4b shows the TGA traces of Priamine^TM^ 1075, Pripol^TM^ 1009, IA, and the bio-based PAs (PA36,IA, and PA36,36) derived using solvent and catalyst-free melt-polycondensation. Greater thermal stability of the bio-based PAs in comparison with starting monomers indicates the formation of PA elastomers (Table 2). Both PAs show traces of similar degradation with a single-stage decay profile, denoting similar thermal decomposition behavior. The initial degradation temperature (T_d5%_) of the bio-based PAs begins at 429.3 °C for PA36,IA, and 427 °C for PA36,36, after which the decomposition accelerates until there is no remarkable residue. The DTG traces of PA36,IA, and PA36,36 samples (Figure 4c) show that the maximal degradation temperature (T_dmax_) occurs at 471 and 470.7 °C. In addition, T_dmax_ is comparable to those of traditional polyamide 11 (PA11), for which T_dmax_ is 470 °C [46]. Thermal decay of linear bio-based PAs starts either in the –CH_2_ group adjacent to –NH or the carbonyl methylene group [47]. Inorganic gases such as water vapor (H_2_O), carbon dioxide (CO_2_), hydrogen cyanide (HCN), and ammonia (NH_3_) are the major products of thermal decay in PAs [48]. The high T_d5%_ and T_dmax_ values of bio-based PA36,IA, and PA36,36 prove their superior resistance to thermal decay.

DSC analysis was employed to study the crystallization and thermal transition behaviors of bio-based PAs. The DSC thermograms of the bio-based PAs are shown in Figure 4d. Due to the existence of multiple dangling chains, the PA36,36 is amorphous in nature, with no T_m_ and T_c_ peaks noticed upon heating and cooling by DSC. For the PA36,IA polymer, the T_m_ peak at 134.6 °C (∆H = 4.3 J g^−1^) was witnessed and the T_c_ peak was not observed because the T_c_ of PA36,IA is mainly affected by the generation of the pyrrolidone ring and the concentration of –NH groups, and also affected by the existence of dangling side chains. First, we observed that it is difficult to determine the T_g_ of all PA elastomers from DSC thermograms. This may be because the sensitivity of the DSC thermogram device is not adequate to detect the T_g_ characteristics of the amorphous domains. Thus, DMA analysis was used instead to evaluate T_g_.

**Figure 5:**
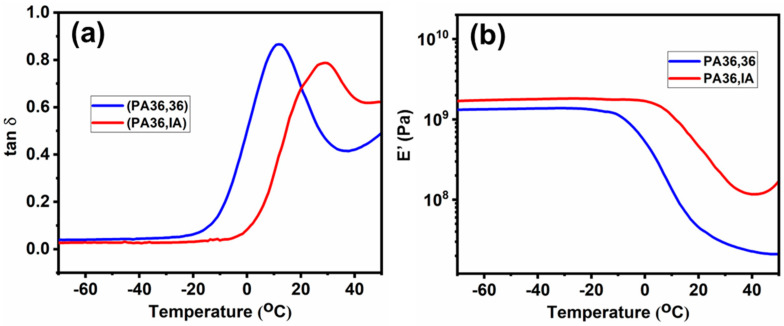
DMA traces of the prepared bio-based PAs: (**a**) tan δ and (**b**) storage modulus.

Figure 5 shows the DMA traces of the PAs as functions of temperature in the range from −75 to 80 °C. There was a distinct variation in the storage modulus (E’) and loss factor (tan δ) of the PA samples (Table 2). DMA analysis shows a higher storage modulus value of PA36,IA, and a slightly lower value for PA36,36 (Figure 5a,b). Furthermore, the small loss factor (dumbing value) of PA36,IA compared to PA36,36 signifies that PA36,IA is a stiffest material and PA36,36 is a more flexible material. The PA elastomers show T_g_, determined as the maximal peak value of tan δ, of 24 °C for PA36,IA and 11.7 °C for PA36,36. The high stiffness of PA36,IA prepared here may be ascribed not only to robust interchain interaction through -NH bonds but also to the PA36,IA network comprising a rigid pyrrolidone ring; however, steric hindrance against interchain H-bonding may be considered by the nonplanar pyrrolidone ring [49]. The T_g_ of PA is associated with the monomers’ chain length and the amide group concentration in the polymer chains. Therefore, longer monomer chains result in greater mobility of PA36,36, meaning it is much softer [50], so the synthesized bio-based PAs possess much lower T_g_ values than the traditional rigid PAs, i.e., PA11 (T_g_ = 68 °C), PA6,6 (T_g_ = 80 °C), and PA6 (T_g_ = 78 °C) [51]. Due to the existence of a rigid pyrrolidone ring in PA36,IA, it exhibits a higher storage modulus value than PA36,36. This may be due to the presence of a hard pyrrolidone ring on the PA36,IA backbone. The low T_g_ value ensures the mobility of the PA elastomers at room temperature, aiding autonomous self-healing [52,53].

### 3.3. Mechanical Properties

**Figure 6:**
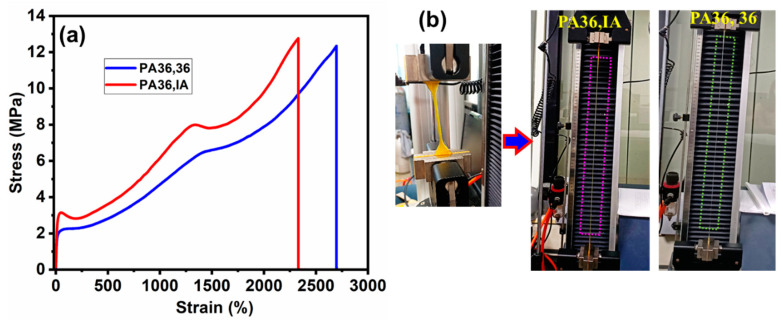
(**a**) Stress–strain curves of PAs and (**b**) stretching photographic images of dumb-bell-shaped PAs.

The descriptive stress–strain (S-S) graphs of PA36,IA and PA36,36 are presented in Figure 6, and the data are summarized in Table 3. The results show an S-S curve shape that is compatible with the thermoplastic elastomers (TPEs), comprising PA-based TPEs [54]. At plastic deformation, when the stress reaches above a certain value, the value of stress linearly increases with the strain, causing plastic deformation. Figure 6a also shows that the stress of each PA continually increases with the strain (strain hardening). The presence of long alkyl dangling chains and a pyrrolidone ring in the PA backbone enhanced the physical cross-linking points, van der Waals forces, and hydrogen bonds, thus effectively improving the strain hardening and leading to the non-linear S-S graphs of PA elastomers. This was reflected in the S-S graphs by the appearance of the evident plastic deformation and yield point. Both the bio-based PAs show notable strain values (Figure 6a,b). However, there was a significant difference in the stress and strain of the PA36,IA and PA36,36 samples. The PA36,IA sample showed significantly lower strain (2330 ± 112%) and higher strength (1.29 ± 0.04 MPa) values in comparison with PA36,36 (strain: 2698 ± 108%, strength: 1.22 ± 0.02 MPa). The rigidity of the pyrrolidone ring in the backbone of PA36,IA may contribute to the higher stress and lower strain values compared to PA36,36. Nonetheless, PA36,IA also shows outstanding stretchability. The entangled and long methylene chains with “hidden lengths” present inside the dynamic physical cross-links of PA36,36 confirm the remarkably higher strain values than those of PA36,IA [55]; when stretched, the long methylene chains detach, and the physical bonds then break, releasing to further extend the hidden chains. By comparison, PA36,IA also exhibits outstanding elongation, which may be due to the existence of long alkyl entangled chains and the formation of the pyrrolidone ring in the polymer backbone structure, which leads to decreased amide group concentration and greater elongation. Additionally, the low M_w_ of PA36,IA is another reason for the high elongation, which facilitates molecular motion and flexibility.

The bio-based PA36,IA and PA36,36 possess the greatest strain values among aliphatic PAs and stretchability that is comparable with that of existing PA-based TPEs. The strain value was ~600% for fatty dimer acid bio-PAs [44], 245 ± 14.4% for PA10,12, 186 ± 12.3% for PA6,10, 197 ± 11.7% for PA10,10 [56], and 350–2200% for fossil-based thermoplastic poly(ether-*b*-amide) elastomers with diverse PA ratios. The strain was also greater than that of other dimer acid-based self-healing rubber-like elastomers (strain ~620%) [30].

### 3.4. Self-Healing Properties of the Bio-Based PA Elastomers

**Figure 7:**
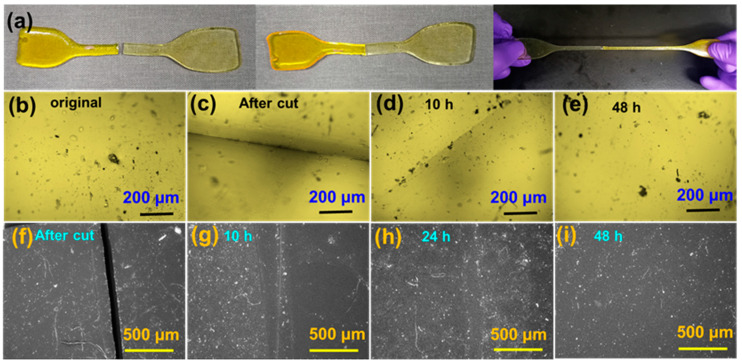
(**a**) Photographs of cut and self-healed PA samples; (**b**–**e**) POM images and (**f**–**i**) SEM images of cut and self-healed PA36,IA samples.

Another interesting property of PA36,IA and PA36,36 is their ability to self-heal. Traditional PA-based TPEs are not self-healing and require alteration to have such characteristics. Both PA36,IA and PA36,36 showed autonomic self-healing properties under ambient temperature, without any alterations or external stimuli. Samples of PA36,IA and PA36,36 were cut into two parts and then reconnected for 1 min before being left for different times at room temperature (~20 °C) (Figure 7a). The photographs in Figure 7a displayed excellent autonomic self-healing capability at ambient temperature. Additionally, the self-healing behavior of bio-based PA elastomers was intuitively monitored by POM (Figure 7b–e and Figure S4a–d) and SEM (Figure 7f–i and Figure S4e–h). As shown in Figure 7b–i, the PA36,IA was first cut into two parts with a 0.4 mm razor blade, after which the two parts were reattached without stress to record the self-healing phenomena using POM and SEM. Results in Figure 7b–e and Figure S4a–d show that the fracture surfaces were sufficiently healed such that the gaps disappeared, thus providing robust proof of the effectiveness of the overall autonomic self-healing process. A similar autonomic self-healing trend was witnessed in PA36,36, for which the POM and SEM images are presented in Figure S4 (Supplementary Material).

**Figure 8:**
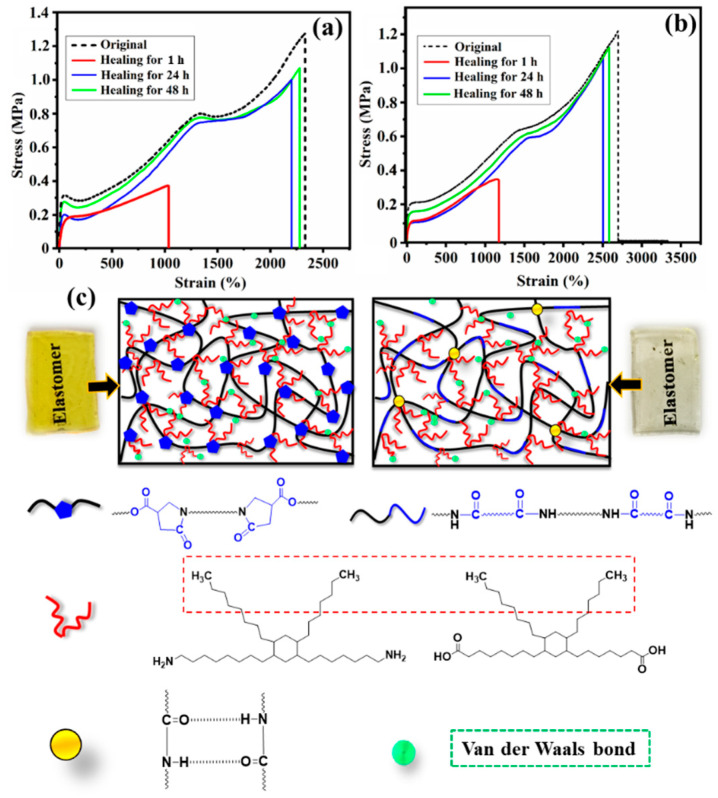
Stress–strain curves of (**a**) PA36,IA, (**b**) PA36,36 PA samples self-healed at room temperature at various times, and (**c**) segment composition of the supramolecular structure of PA elastomers.

Next, the self-healing attributes of the bio-based PA elastomers were further elaborated by S-S traces. As demonstrated in Figure 8a,b, the mechanical attributes of the PA elastomers were recovered with increasing self-healing time. After 1 h, the PA36,IA and PA36,36 samples were stretched to 1029 ± 217% and 1196 ± 195%, respectively, whereas, after 48 h, they were stretched to 2265 ± 156% and 2638 ± 147%, which is ~97% of the average strain value of the original PA specimens, before being cut into two parts (Table 3). The strain value of self-healed PA36,IA and PA36,36 samples showed a self-healing ability of 44% and 44%, respectively, after 1 h, and 97% and 98% after 48 h. This autonomic self-healing ability is remarkable for fully bio-based polymer materials. The self-healing attributes of PAs were compared. With an identical healing time (48 h), the healing ability of the two PAs was almost identical. Interestingly, PA36,IA, which contains a hard pyrrolidone fraction, also exhibited a healing ability of 97%. The probable cause is a decrease in M_w_ of PA36,IA compared to PA36,36, which enhances the intermolecular segment migration.

Compared with a number of previous studies on bio-based self-healing polymers [57,58], PA36,IA and PA36,36 displayed advantages in terms of more remarkable healing ability, shorter healing time, and no need for external stimuli.

As illustrated in Figure 8c, the fatty acid monomer of PA36,IA and PA36,36 consists of dangling side chains with several alkyl groups. After polycondensation, the key long methylene and dangling chains in the PA form numerous van der Waals bonds amid the methylene groups similar to a supramolecular network, which was found to be robust, reversible, and dynamic [59]. After physical damage to the PA specimens, the van der Waals bonds break, and the attached chains become free dangling chains leading to an entropic rise [60]. When the two broken PA elastomer specimen parts are brought back together, the main and dangling chains spread from one part to the other, and again form robust van der Waals forces/interactions between the elastomer chains; thus the PA elastomer self-heal until the entropy of the system returns to equilibrium [61].

### 3.5. Viscoelastic Properties

The viscoelastic features play a key role in the foaming process. Rheological analyses were carried out at 150 °C to evaluate the viscoelastic attributes of bio-based PAs. Both of the bio-based PAs are thermostable up to 420 °C, which means the PAs do not thermally decompose during viscoelastic experiments. Complex viscosity (η*), storage modulus (G’), loss modulus (G”), and loss factor (tan δ) of PA36,IA and PA36,36 were derived as a function of angular frequency (ω). Both the elastomers acted in a similar way and the η* values decreased, whereas the G” and G’ values increased with increasing ω. The rheological properties of molten polymers strongly depend on M_w_ [62].

A shear-thinning behavior of bio-based PA elastomers is witnessed in Figure S5a (Supplementary Material). The η* values of PA36,IA declined compared to those of PA36,36, resulting from the reduction in M_w_. The greater η* values can improve the melting strength and better inhibit the foam cell rapture throughout the cell growing stage during the scCO_2_ foaming process. Figure S5b showed the associations of G’ and ω. It can be seen that the G’ curve of PA36,36 is higher than that of PA36,IA due to its higher M_w_. A similar trend was observed for G” values (Figure S5c). The definition of tan δ is the contribution ratio (G”/G’) at a specific value of ω. For the PA36,IA elastomer, the increasing trend in tan δ is observed in Figure S5d. It is shown that the melting elasticity deteriorates and the melting viscosity loss progressively improves. Polymers with higher melting strength are anticipated to improve the foamability.

### 3.6. Water Uptake Study

**Figure 9:**
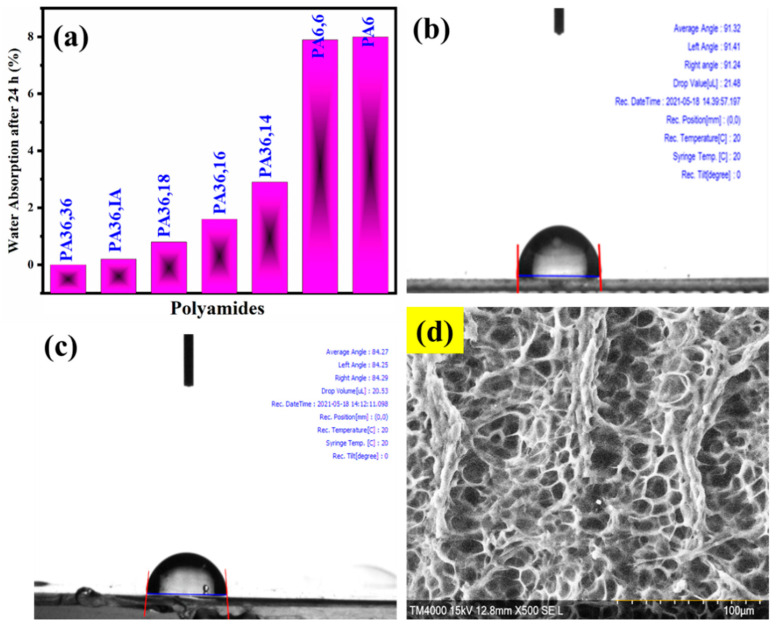
(**a**) Water absorption traces of the synthesized PA elastomers and previously reported different chain-length PAs; water contact angle traces of PA36,IA (**b**) and PA36,36 (**c**); SEM image of PA36,36 foam (**d**).

Figure 9a displays the percentage of water uptake of PA36,IA and PA36,36 after being submerged in water for 24 h compared with other PAs reported in previous literature [51]. PA36,IA displays a water absorption value of 0.2%, and PA36,36 displays water absorption of zero percent after 24 h of water submersion, representing hydrophobicity. However, compared to other traditional PAs, our integrated PA36,IA also exhibits a lower water absorption value, indicating that PA36,IA is significantly less sensitive to water. A lower ratio of –NH- linkages in the PA chains contributes to reduced water absorption. Based on the comparatively low –NH– ratio of PA36,36, this polymer has relatively zero water absorption properties and hydrophobicity. The origin of the monomer is another cause of the hydrophobicity of PA36,IA and PA36,36. The Priamine^TM^ 1075 and Pripol^TM^ 1009 monomers are derived from long-chain fatty acids, which are generally hydrophobic. Priamine^TM^ 1075 and Pripol^TM^ 1009 both comprise non-polar hydrocarbon chains and polar chain ends, which control water uptake. The water contact angle (θ) measurement was utilized to affirm the hydrophobicity of PAs (Figure 9b,c). Polymers with θ > 90° are defined as hydrophobic materials. The θ values of PA36,IA and PA36,36 were 84° and 91°. PA36,36 has a higher θ value compared to other traditional PAs such as PA6, PA66, and PA6,10 [63].

### 3.7. Absorption Performance

**Figure 10:**
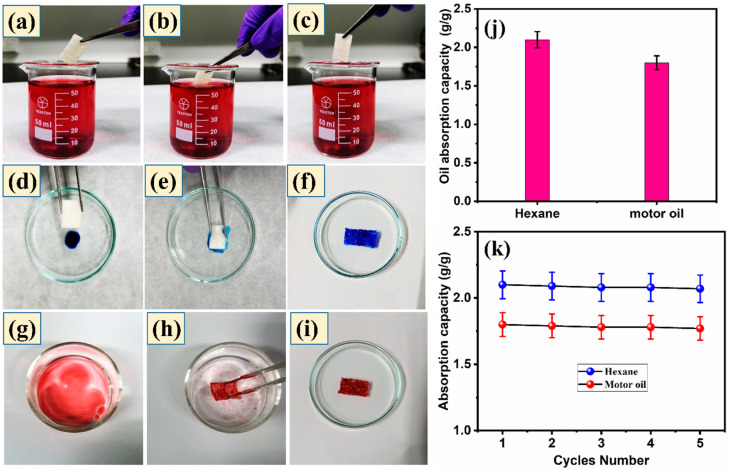
(**a**–**c**) Images of the PA36,36 foam being immersed in water; (**d**–**f**) absorbing dyed hexane on the glass surface; (**g**–**i**) absorbing dyed hexane on top of water; (**j**) absorption capacity of PA36,36 foam towards hexane and motor oil; and (**k**) cyclic absorption traces for PA36,36 foam.

In this work, PA36,36 only exhibited higher M_w_ and hydrophobic attributes compared to PA36,IA. Therefore, as a proof of concept, PA36,36 was selected as the substrate for the production of foam for the oil absorption study. For this test, PA36,36 foams were prepared by the scCO_2_ foaming process. The prepared foam shows a microporous cellular structure (Figure 9d). To ensure the use of PA 36,36 foam in the selected oil absorption, the wettability test was first performed. After immersion in water, no water was seen to be absorbed by the PA36,36 foam (Figure 10a–c, Movie S1 in Supplementary Material). Accordingly, the PA36,36 foam has excellent potential for oil absorption applications. To test the oil absorption behavior of PA36,36 foam, dyed oils (hexane and motor oils dyed using Oil Red O dye and Oil Blue A dye) were used. Figure 10d-f shows a piece of PA36,36 foam entirely absorbed the dyed oils from the surface of the glass. When in contact with the dyed oils, the PA36,36 foams absorbed the oils quickly and entirely. Moreover, PA36,36 foam is expected to absorb oils easily from oil/water mixtures (Figure 10g–i). Hence, PA36,36 foam was also used to selectively absorb dyed hexane on top of the water. This absorption process is displayed in Movie S2 (Supplementary Material), in which PA36,36 foams can be seen to absorb the dyed hexane rapidly from the water. In addition, no water was discovered in the PA36,36 foam, and no dripping of absorbed oils was found during the absorption process, indicating the high selectivity of the oils. The absorption characteristics of PA36,36 foam were also estimated by measuring the weight of oil, i.e., the ratio of the mass of absorbed oil to the PA36,36 foam. The absorption capacity of hexane and motor oils of PA36,36 foam was found to be 2.1 and 1.8 g/g, respectively, depending on the viscosity and density of the oils (Figure 10j). Moreover, it is vital to examine the consistency of the absorption capacity in repeated usage. The PA36,36 foam was deployed to absorb hexane and motor oil for five cycles, and the results are presented in Figure 10k. It is shown that the absorption capacity of PA36,36 foam was the same when the motor oil and hexane were absorbed. Due to its eco-friendliness, the bio-based PA36,36 foam holds significant potential for utilization as an oil absorber in practical use.

## 4. Conclusions

In summary, highly stretchable, autonomous self-healing, hydrophobic, and wholly renewable PA-based thermoplastic elastomers were successfully prepared by one-pot green melt polycondensation under catalyst and solvent-free conditions. The M_w_ values of the elastomers were above 30,000 g mol^−1^, and ^1^H NMR, ^13^C NMR, and FTIR verified their molecular structures. PA36,IA was found to have T_m_ of 134.6 °C, whereas PA36,36 showed an amorphous nature, and both elastomers were found to have T_d5%_ above 420 °C. The elastomers showed low T_g_ values of 24 °C for PA36,IA and 11.7 °C for PA36,36, which aids the autonomous self-healing capability at ambient temperatures (healing efficiency of ~97% in 48 h). The stress–strain (S-S) test revealed that the elastomers had excellent strain values (PA36,IA: 2330 ± 112% and PA36,36: 2698 ± 108%). These elastomers consisted of an abundance of van der Waals forces from long entangled alkylene chains, manifold dangling chains, and intermolecular diffusion, which led to better self-healing behavior. Interestingly, the PA36,36 elastomer is hydrophobic, as confirmed by its water contact angle (θ = 91°) and the water absorption study. This is a unique attribute compared to traditional water-absorbing PAs. The high M_w_ and special hydrophobic attributes of the PA36,36 elastomer permit the fabrication of PA36,36 foam. SEM images of the PA36,36 foam indicate a microporous cellular structure, which assists in better absorption capacity and greater stability in the repeated use for selective oil absorption. This novel generation of fully renewable elastomers can be rated as a class of renewable eco-friendly self-healing material, and shows significant potential to replace fossil-based alternative products.

## Figures and Tables

**Table 1:** The M_w_ and M_n_ of the synthesized PAs.

Samples	M_w_ (g mol^−1^)	M_n_ (g mol^−1^)	Ð
PA36,IA	30,889	19,262	1.60
PA36,36	32,938	21,045	1.57

**Table 2:** Thermal degradation and glass transition temperatures of bio-based PA elastomers.

Samples	T_dmax_ (°C)	T_g_ (°C)
IA	217.0	-
Priamine^®^1075,	356.6	-
Pripol^TM^1009	385.3	-
PA36,IA	471.0	24.0
PA36,36	470.7	11.7

**Table 3:** Tensile properties of fully bio-based PA elastomers.

Sample	Strength (MPa)	Strain (%)
PA36,IA (original)	1.29 ± 0.04	2330 ± 112
PA36,36 (original)	1.22 ± 0.02	2698 ± 108
PA36,IA (healing for 1 h)	0.39 ± 0.05	1029 ± 217
PA36,IA (healing for 24 h)	1.00 ± 0.11	2206 ± 170
PA36,IA (healing for 48 h)	1.08 ± 0.06	2265 ± 156
PA36,36 (healing for 1 h)	0.35 ± 0.09	1196 ± 195
PA36,36 (healing for 24 h)	1.07 ± 0.13	2502 ± 147
PA36,36 (healing for 48 h)	1.11 ± 0.15	2648 ± 125

## Supplementary Materials

The following are available online at https://www.mdpi.com/article/10.3390/polym13183089/s1, The data contains the files are following: Polymerization reactor image, M_w_, M_n_ and polydispersity, UV-Visible spectra, ^13^C NMR, DMA analysis, POM, SEM, and rheological properties.
