# Supplementary material for: Highly Stretchable Fully Biomass Autonomic Self-Healing Polyamide Elastomers and Their Foam for Selective Oil Absorption
Source: Polymers (Basel). 2021 Sep 13;13(18):3089. doi: 10.3390/polym13183089 (PMC8468103; doi:10.3390/polym13183089)
Supplement: Supplementary file 1 [file polymers-13-03089-s001.zip › polymers-1356043-supplementary.pdf]

Highly Stretchable Fully Biomass Autonomic Self-Healing Polyamide Elastomers and Their Foam for Selective Oil Absorption

Palraj Ranganathan, Chin-Wen Chen*, Syang-Peng Rwei*

Institute of Organic and Polymeric Materials, Research and Development Center of Smart Textile Technology, National Taipei University of Technology,

No. 1, Sec. 3, Chung-Hsiao East Road., Taipei, 10608, Taiwan (R.O.C.)

Corresponding Author:

Prof. Syang-Peng Rwei: f10714@ntut.edu.tw

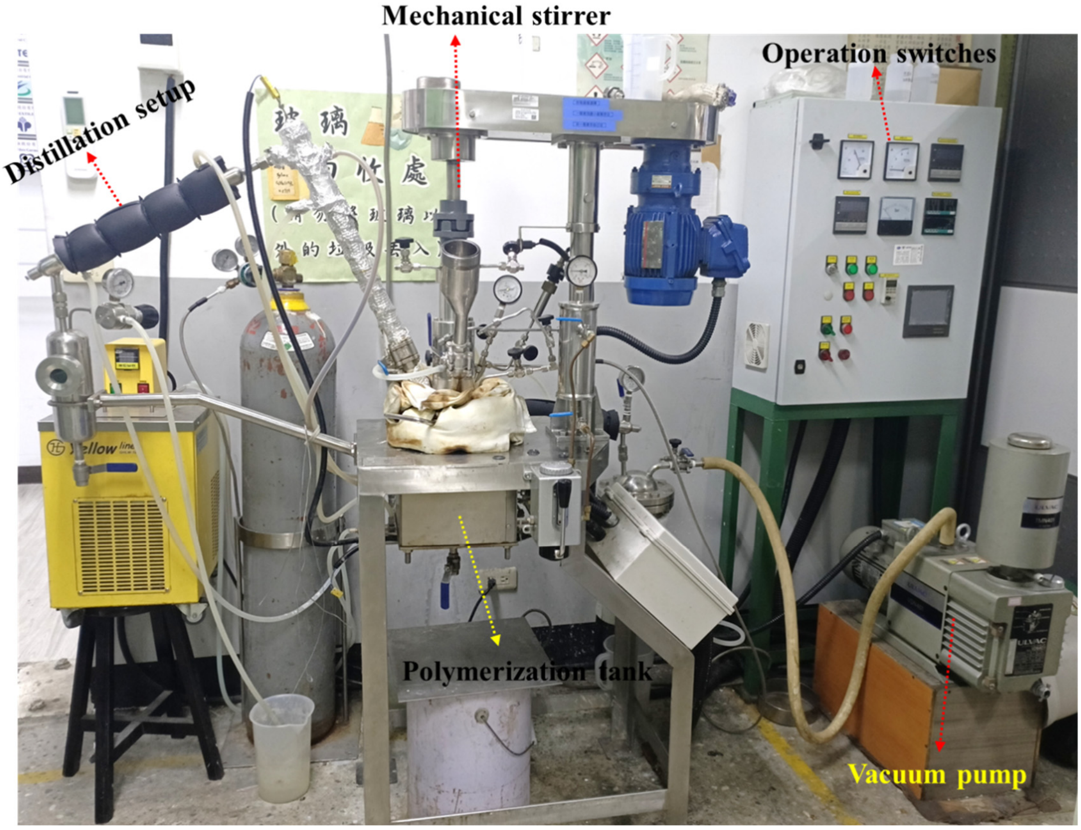

Figure S1. Polymerization reactor used in this work for the synthesis of bio-based PA elastomers.

Synthesis of PA36,36 Thermoplastic Elastomer

An equimolar amount of Pripol^{TM}1009 (1000 mmol, 570 g) and Priamine^{TM}1075 (1000 mmol, 570 g) were weighed and introduced into a 2 L polymer reactor equipped with a central mechanical stirrer, nitrogen gas in and outlet, and a distillation setup thermometer, and a vacuum pump connection. To prevent excess foaming or bubbles, the reaction temperature was gradually raised by 20 °C every 25 min until attaining 220 °C. Subsequently, the polymerization was performed for 12 h at 220 °C. After 12 h, the polymerization reaction was continued for 1 h under reduced pressure (P = 0.7 kPa). The final product was transferred to a storage container from the reactor. The yield of the final product reaches 97%.

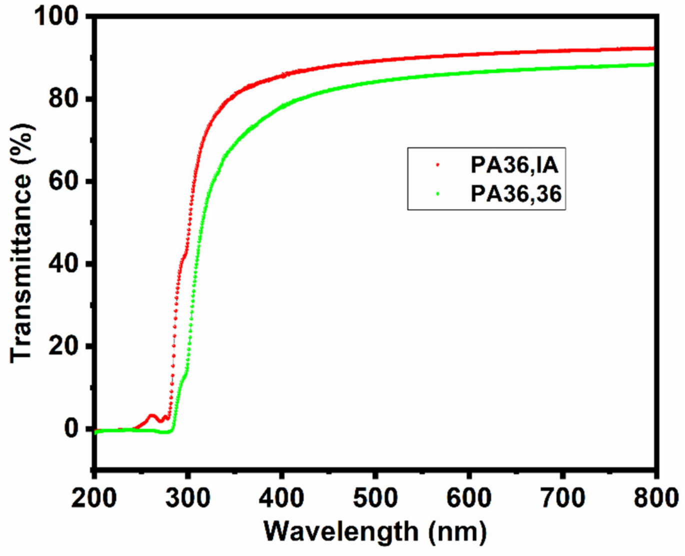

Figure S2. UV-Vis spectra of the PA elastomers.

UV-visible spectroscopy was performed to inspect the transparency of the prepared PA elastomer films, and the traces are bestowed in Figure S2. PA36,36 films display great transmittance, 92%, and 85% respectively, in the UV region 400-800 nm, and its cut-off wavelengths are originated at 250 nm, which is better than those of UPILEX® and KAPTON®. On the other hand, PA36,IA films display transmittance, 88%, and 78% respectively, in the UV region 400-800 nm, and its cut-off wavelengths are originated at 250 nm.

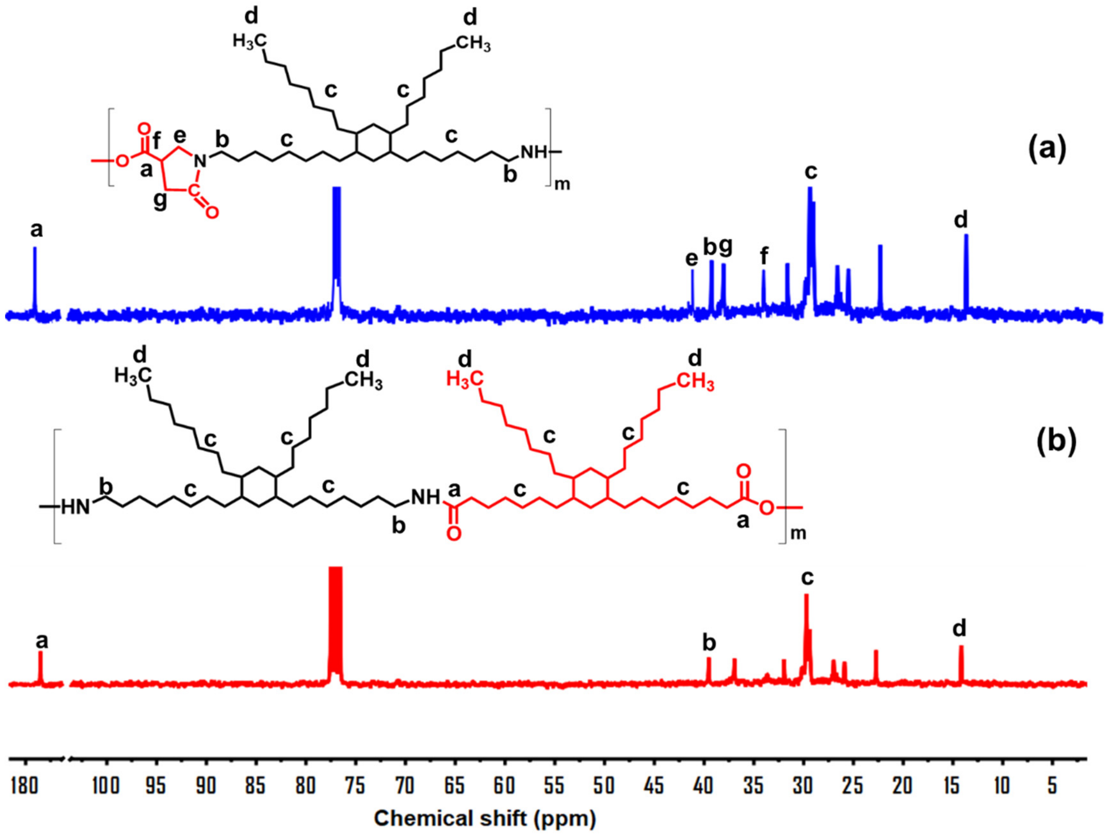

Figure S3. ^{13}C NMR Spectra of (a) PA36,IA and (b) PA36,36.

In the ^{13}C NMR spectra of PA36,IA (Figure S3a), the chemical shifts are allocated as follow: ^{13}C in –CO group from –NH- linkage detect at 175.74 ppm, in –CH_2 adjacent to –NH linkage at 39.81 ppm, in the –CH_2 middle of aliphatic polymer chains detect at 30.1 ppm, in the –CH_3 dangling chains detect at 14.36 ppm, in -CH_2 adjacent the -NH linkage in the pyrrolidone ring detected at 39.81 ppm, in -CH_2 adjacent the pyrrolidone ring –CO linkage detected at 34.12 ppm and 38.33 ppm.

In the ^{13}C NMR spectra of PA36,36 (Figure S3b), the chemical shifts are allocated as follow: ^{13}C in –CO group from –NH- linkage detect at 175.74 ppm, in –CH_2 adjacent to –NH linkage at 39.81 ppm, in the –CH_2 middle of aliphatic polymer chains detect at 30.1 ppm, in the –CH_3 dangling chains detect at 14.36 ppm.

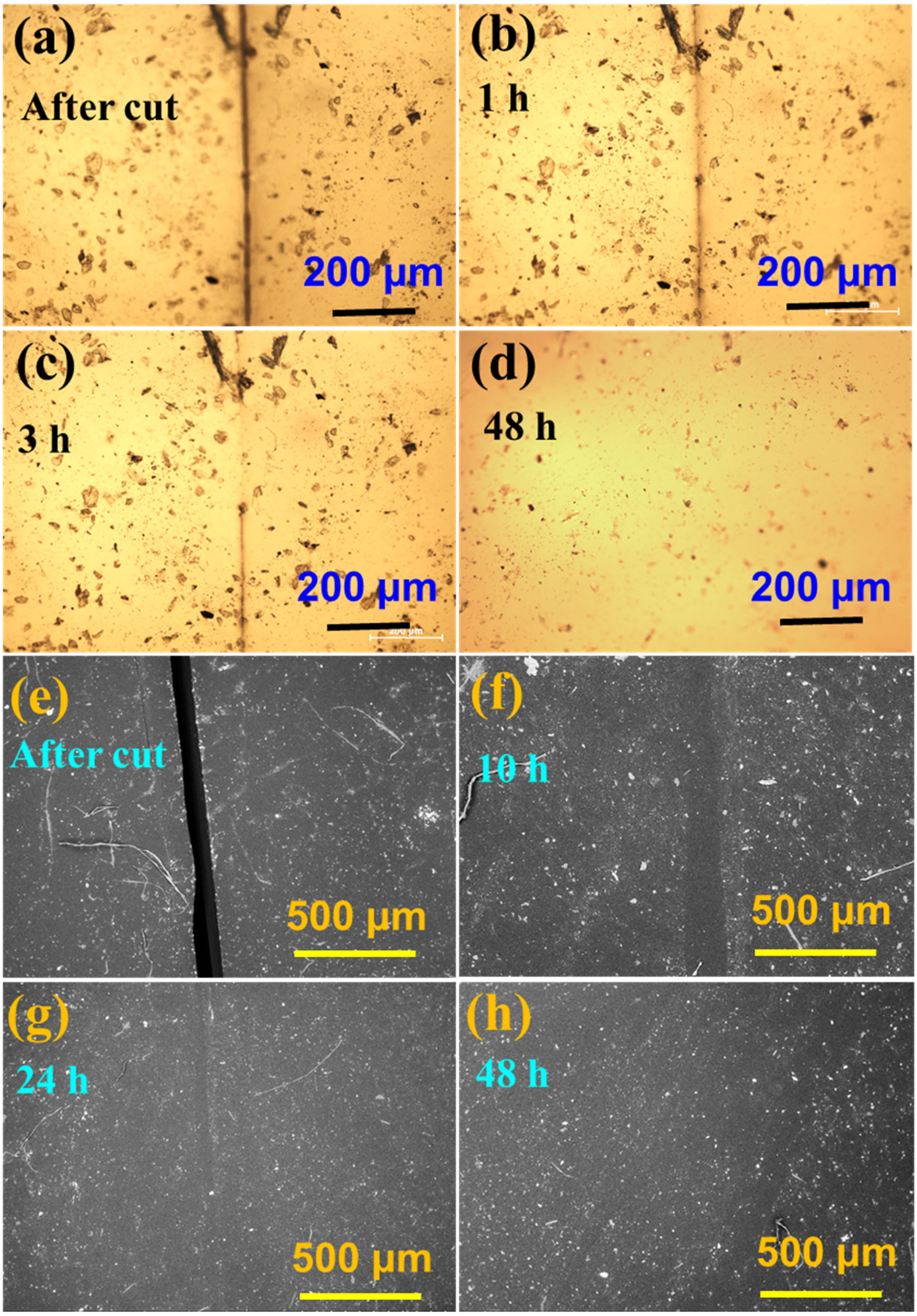

Figure S4. POM (a-d) and SEM (e-h) images of cut and self-healed PA36,36 elastomer samples.

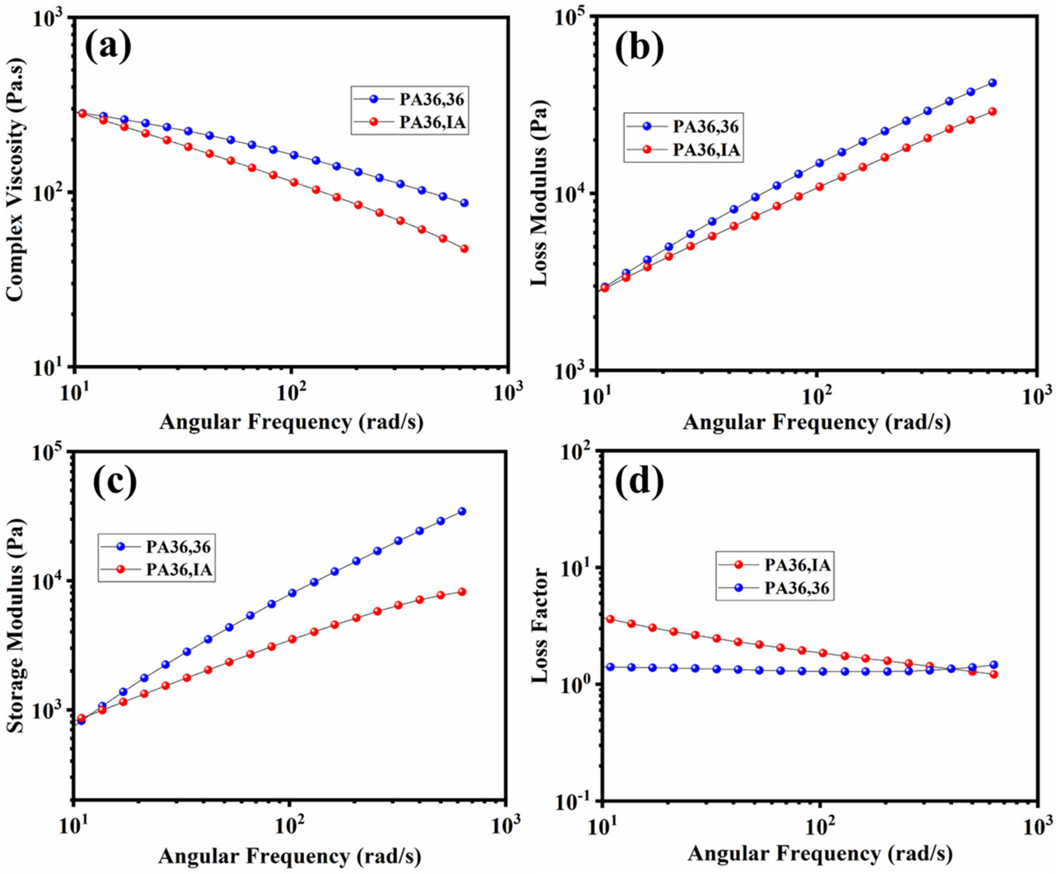

Figure S5. Rheological properties of PA elastomers: (a) complex viscosity (η*), (b) loss modulus (G"), (c) storage modulus (G'), and (d) loss factor (tan δ).

Videos

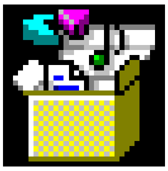

Movie-1.mp4

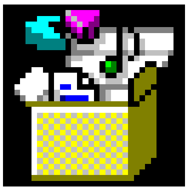

Movie-2.mp4
